# Resveratrol promotes trophoblast invasion in pre‐eclampsia by inducing epithelial‐mesenchymal transition

**DOI:** 10.1111/jcmm.14175

**Published:** 2019-02-01

**Authors:** Yanfen Zou, Shuhong Li, Dan Wu, Yetao Xu, Sailan Wang, Ying Jiang, Fang Liu, Ziyan Jiang, Hongmei Qu, Xiang Yu, Xiaoli Wang, Yuanli Wang, Lizhou Sun

**Affiliations:** ^1^ Department of Obstetrics and Gynecology Affiliated Yantai Yuhuangding Hospital of Qingdao University Yantai Shandong Province China; ^2^ Department of Obstetrics and Gynecology First Affiliated Hospital of Nanjing Medical University Nanjing Jiangsu Province China; ^3^ Department of Obstetrics, Gynecology & Reproductive Sciences Yale Stem Cell Center, Yale University School of Medicine New Haven Connecticut; ^4^ Department of Obstetrics, Women’s Hospital Zhejiang University School of Medicine Hangzhou Zhejiang P.R. China; ^5^ Department of Cardiology, Beijing Anzhen Hospital Capital Medical University Beijing China; ^6^ Department of General Surgery Affiliated Yantai Yuhuangding Hospital of Qingdao University Yantai Shandong Province China

**Keywords:** EMT, pre‐eclampsia, resveratrol, spiral artery remodelling, Wnt/β‐catenin

## Abstract

Impairment spiral arteries remodelling was considered to be the underlying cause of pathogenesis of pre‐eclampsia (PE). Resveratrol (RE) was reported that it could modulate cellar phenotype to ameliorate diverse human diseases. However, the biological function of RE in PE remains poorly understood. In this report, we investigated the effect of RE on trophoblast phenotype both in vivo and in vitro. We conducted MTT and transwell assays to explore cell proliferation and invasion events in HTR‐8/SVneo. In mice model, the clinical characteristics of PE were established through the injection of NG‐nitro‐l‐arginine methyl ester (L‐NAME). Furthermore, related experiments were performed to detect cellar phenotype‐associated signalling pathway, including epithelial‐mesenchymal transition (EMT) and Wnt/β‐catenin. Cell assays indicated that RE could increase trophoblasts migration and invasion. In addition, hypertension and proteinuria were markedly ameliorated by RE compared with the controls in PE mice model. Moreover, treatment by RE in trophoblasts or in PE model, we found that RE activated EMT progress through the regulation of E‐cadherin, β‐catenin, N‐cadherin, vimentin expression, and further altered the WNT‐related gene expression, including WNT1, WNT3 and WNT5B. Our findings demonstrated that RE might stimulate the invasive capability of human trophoblasts by promoting EMT and mediating the Wnt/β‐catenin pathway in PE.

## 1 INTRODUCTION

Pre‐eclampsia (PE), a devastating multi‐system syndrome, occurs in approximately 10% of pregnancies worldwide.^1^ It is the major trigger leads to pregnancy‐associated mortality and serious damage to the spleen, liver, kidney and many other organs. Previous evidence have established that diversified factors may involve in the occurrence and development of PE, such as impaired spiral artery remodelling, oxidative stress and oxygen dysregulation.^2^, ^3^, ^4^ Among these factors, aberrant trophoblast invasion, which contributed to impaired spiral artery remodelling, is considered as the underlying cause of pathogenesis in PE.^5^ The acquisition of invasive capabilities by trophoblasts is determined by the turnover of cell‐cell junctions, degradation of the cell matrix and activation of pathways that control cytoskeletal dynamics.^6^

Recently, epithelial‐mesenchymal transition (EMT) has been known to play an essential role in the regulation of cell migration and invasion. It is characterized by the breakdown of cell‐cell adhesion, loss of epithelial phenotypes and cell depolarization, which accelerate progression in multiple diseases, especially cancer.^7^, ^8^ EMT is important in the oncogenesis, metastasis and drug resistance of cancer progression.^9^ During pregnancy, the acquisition of invasive phenotypes by extravillous trophoblast (EVT), which is involved in the pathogenesis of PE, has been proposed to be associated with EMT process.^10^ However, the underlying regulatory mechanisms of EMT in trophoblasts in the pathogenesis of PE are still poorly understood.

Resveratrol (trans‐3,4,5‐trihydroxy‐stilbene; RE), a traditional Chinese medicine, is known as a naturally occurring compound with potent biological properties.^11^, ^12^, ^13^ It is found in plants, such as grapes, giant knotweed, peanuts and mulberries.^14^ RE exerts protective effects against various types of cancers^15^, ^16^ partly by modulating cancer cell invasion,^17^ and also can reduce platelet aggregation in haematological system diseases.^18^, ^19^ Moreover, long‐term basic research and clinical research have confirmed that RE exerts protective effects against cardiovascular and cerebrovascular diseases (such as hypertension, ventricular hypertrophy, atherosclerosis, myocardial infarction and arrhythmias), inflammatory lesions and diabetes.^20^, ^21^ Also, many studies have reported that RE significantly ameliorates an oxidative‐stress reaction in pregnant model with hypertension and proteinuria.^22^ However, the underlying molecular mechanisms of RE remain to be elucidated.

Hence, PE mice model was first established through the intraperitoneal injection of NG‐nitro‐l‐arginine methyl ester (L‐NAME), an inhibitor of nitric oxide (NO) synthase.^23^ We focused on that RE significantly improved the clinical indicators of PE in vivo. Similarly, cell assays verified that RE could promote trophoblasts invasion and proliferation, further ameliorating the impairment of spiral artery remodelling in PE. Additionally, associated mechanistic exploration demonstrated that RE might stimulate the invasive capability of human trophoblasts by promoting EMT process and mediating the Wnt/β‐catenin pathway, further suggested that RE might serve as a latent therapeutic agent for PE.

## 2 MATERIALS AND METHODS

### 2.1 Ethics statement

The research involving humans was performed with prior approval from the Ethics Committee of the First Affiliated Hospital of Nanjing Medical University. All participants provided a written informed consent prior to the study. All animal experiments were performed with approval from the Institutional Animal Ethics Committee and performed in accordance with the guidelines of the Committee for the Purpose of Control and approved by the Care of Experimental Animals Committee of Nanjing Medical University Ethics Committee. All efforts were made to minimize suffering in experimental animals.

### 2.2 Animal models

A mouse model of PE was established through the intraperitoneal injection of the eNOS inhibitor L‐NAME (125 mg/kg body weight). Pregnant adult female albino Wistar mice (weighing 200‐250 g) were divided immediately and randomly into three groups (n = 16 per group): control, L‐NAME, and L‐NAME + RE groups. RE was administered intragastrically at a dose of 20 mg/kg/day. On the gestational day (GD) 18.5, the mice were killed and urine, blood, placenta and uterine samples were collected. Blood pressure was detected using a programmed electronic sphygmomanometer (BP‐98A; Softron, Tokyo, Japan) by the tail‐cuff method at 8.30 am every morning during pregnancy. Urine samples were collected on GD 0.5, 4.5, 9, 13.5 and 18.5 and stored at −80°C. Urinary levels of albumin were detected on the same GDs (Behoite, Beijing, China and Cayman, MI, USA).

### 2.3 Cell culture and treatment

In this study, HTR‐8/SVneo Cells were provided by Dr Charles Graham, Queen's University, Canada. Human umbilical vein endothelial cells (HUVEC)‐C were obtained from the Type Culture Collection of the Chinese Academy of Sciences (Shanghai, China). HTR‐8/SVneo and HUVEC cells were cultured in RPMI 1640 and ECM (KeyGEN, Nanjing, China), respectively, which were supplemented with 10% foetal bovine serum (FBS; Gibco BRL, Invitrogen, Carlsbad, CA, USA), 100 U/mL penicillin and 100 mg/mL streptomycin (Invitrogen). The cells were maintained at 37°C in a humidified atmosphere with 5% CO_2_. HTR‐8/SVneo cells were seeded in six‐well culture plates overnight and exposed to the following treatments for 24 hours: no treatment (normal group), 20 mmol/L DOX (Sigma, USA) (DOX group),and 20 mmol/L DOX + 100 µmol/L RE (Selleckchem, USA) (DOX+RE group).

### 2.4 RNA extraction and qPCR assays

Total RNA was extracted from approximately 0.1 g of placenta tissues or from trophoblasts using TRIzol reagent (Invitrogen Life Technologies). A reverse transcription kit (Takara) and Power SYBR Green (Takara) were used for cDNA synthesis and amplification respectively. The expression of genes regulating migration, invasion, angiogenesis and EMT‐related factors were detected by SYBR Green qRT‐PCR. The expression of glyceraldehyde‐3‐phosphate dehydrogenase (GAPDH) was used for normalization. The primers used were as follows: E‐cadherin (forward: 5‐TCCCATCAGCTGCCCAGAAA‐3, reverse: 5‐TGACTCCTGTGTTCCTGTTA‐3); N‐cadherin (forward: 5‐CCCTGCTTCAGGCGTCTGTA‐3, reverse: 5‐TGCTTGCATAATGCGATTTCACC‐3); Snail (forward: 5‐GACCACTATGCCGCGCTCTT‐3, reverse: 5‐TCGCTGTAGTTAGGCTTCCGATT‐3); β‐catenin (forward: 5‐CTCAGGACAAGGAAGCTGCAGAAGC‐3, reverse: 5‐CAAGGCATCCTGGCCATATCCA‐3); Vimentin (forward: 5‐TCTACGAGGAGGAGATGCGG‐3, reverse: 5‐GGTCAAGACGTGCCAGAGAC‐3); MMP‐2 (forward: 5‐CGTCTGTCCCAGGATGACATC‐3, reverse: 5‐TGTCAGGAGAGGCCCCATAG‐3); MMP‐9 (forward: 5‐TGGGCAGATTCCAAACCTTT‐3, reverse: 5‐TCTTCCGAGTAGTTTTGGATCCA‐3); VEGF (forward: 5‐CTGCTGTCTTGGGTGCATTGG‐3, reverse: 5‐CACCGCCTCGGCTTGTCACAT‐3); sFlt‐1 (forward: 5‐AGGAGATGCTCCTCCCAAA‐3, reverse: 5‐GTGCAGGGATCCTCCAAAT‐3); Ang‐I (forward: 5‐CAACAACAGTGTCCTTCAGAA‐3, reverse: 5‐CTTTAGTGCAAAGATTGACAAGGTT‐3); Ang‐II (forward: 5‐GTCCACCTGAGGAACTGTCT‐3, reverse: 5′‐TTGTGACAGCAGCGTCTGTA‐3); GAPDH (forward: 5‐GACTCATGACCACAGTCCATGC‐3, reverse: 5‐AGAGGCAGGGATGATGTTCTG‐3). An ABI 7500 system was used to carry out the qPCR and data collection.

### 2.5 Western blotting analysis

Proteins were extracted from HTR‐8/SVneo cells or from mice placentas, treated with RIPA Protein lysis buffer (Beyotime), and supplemented with protease inhibitor cocktail (Roche) and phenylmethylsulfonyl‐fluoride (Roche). Protein concentration was measured using the Bradford assay. Next, samples containing 50‐100 µg of protein were separated by 10% SDS‐PAGE. The protein on the gel was then transferred onto 0.22‐mm polyvinylidene difluoride membranes (Sigma). Specific antibodies against E‐cadherin, N‐cadherin, snail, vimentin, β‐catenin, MMP‐2, MMP‐9, WNT1, WNT3 and WNT5B were all purchased from Cell Signaling Technology (MA, USA) and used as primary antibodies (1:1000). The secondary antibody used was horseradish peroxidase‐conjugated goat anti‐rabbit IgG or goat anti‐mouse IgG (1:1000; Beijing Zhong Shan Biotechnology Co., Beijing). GAPDH (1:1000; Santa Cruz, CA, USA) was used as a control. We used the Quantity One software (Bio‐Rad) to quantify the intensity of the protein bands.

### 2.6 Immunofluorescence

The placental tissues were removed from the rats and immediately fixed in 4% paraformaldehyde according to a standard protocol. HTR‐8/SVneo cells were also fixed through the same procedure. Specific polyclonal antibodies against E‐cadherin (BD, USA), N‐cadherin (BD), β‐catenin (BD), vimentin (BD) and snail (Cell Signaling Technology) at a concentration of 1:100 were used as primary antibodies. TRITC‐labelled anti‐Rabbit IgG (1:200; Sigma) was used as a secondary antibody. An Olympus BX51 microscope (Olympus Optical, Tokyo, Japan) was used to capture the immunofluorescence images.

### 2.7 Cell invasion assays

Cell invasion assays were performed as previously reported by Xu et al.^24^ The RE‐treated HTR‐8/SVneo cells were suspended in RPMI 1640 medium containing 1% FBS and subsequently seeded at a density of 5×10^5^ cells/well to the upper well of a transwell chamber (Millipore, Billerica, MA, USA) with a membrane pore diameter of 8 µm. Medium containing 10% FBS, as a chemo‐attractant, was immediately placed in the lower chamber. After 24 hours of incubation, the cells on the upper surface were removed, whereas the cells on the lower surface were fixed and stained with 0.1% crystal violet. Finally, the number of migrated cells was calculated under a digital microscope in five random fields for each sample.

### 2.8 Tube formation assay

Tube formation assay was performed as previously described by Xu et al.^25^ To determine the potential of blood vessel formation in spiral artery remodelling in vitro, this assay was introduced to simulate in HTR‐8/SVneo (or HUVEC) cells treated with RE. Transwell cell culture inserts (12 mm diameter, 0.4 mmol/L pore size; Corning, Fisher Scientific UK Ltd., Loughborough, UK) were coated with 100 µL of growth factor‐reduced Matrigel (BD Biosciences, Oxford, UK) and allowed to set at 37°C for at least 30 minutes. Next, a total of 2.5×10^4^ cells was seeded on the upper layer of the chamber and incubated at 37°C with 5% CO_2_ for 24 hours. Then, the inserts were washed three times to remove scattered or the bridge‐like cells that did not form networks. Next, the cells were fixed by methanol for 30 minutes and stained with crystal violet. Finally, images from each well were captured under a light microscope. The number of cell‐cell protracted contacts and the length of tubes were counted as the number of capillary‐like networks present in each field.

### 2.9 Statistical analysis

All data are expressed as the mean ± SD. All statistical analyses were performed with the SPSS statistical software package (SPSS Inc, Chicago, IL, USA). Paired and unpaired Student's *t* tests were used to compare the results of two groups and ANOVA was used to compare the data of more than two groups. *P* < 0.05 was considered statistically significant.

## 3 RESULTS

### 3.1 PE clinical phenotypes were ameliorated by RE in mice model

**Figure 1:**
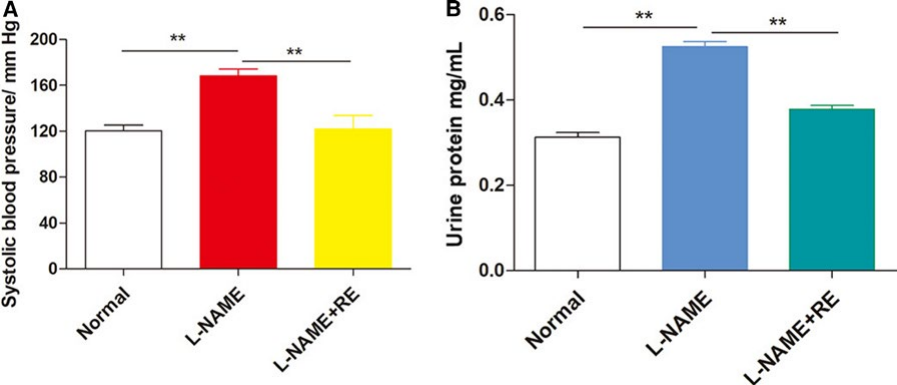
RE ameliorated many PE phenotypes in PE mice model. The L‐NAME group exhibited significantly higher blood pressure and urinary protein level than the control and L‐NAME + RE groups. **P < 0.01

To explore the role of RE in PE, we first used L‐NAME to simulate a PE mice model. After treatment with RE, the related clinical indicators, including systolic blood pressure, proteinuria, foetus number, foetus birth weight, placental weight and external malformations, were measured and observed in all groups (n = 16 per group) (Table 1). There are no significant differences in foetus number, foetus birth weight, placental weight and external malformations between the groups. The L‐NAME + RE groups exhibited significantly lower BP and urine protein level compared with the L‐NAME group (Figure 1A and B), indicating the successful establishment of a PE model using L‐NAME and the ameliorative effects of RE on PE clinical phenotypes.

**Table 1:** Clinical Parameters of pregnant mice among three groups

Parameters	Control (n = 16)	L‐NAME (n = 16)	L‐NAME+RE (n = 16)	*P* a‐value
Systolic blood pressure, mm Hg	124.39 ± 11.30	171.22 ± 10.41	130.07 ± 15.62	<0.05
Protein mg/mL (24 h)	0.31697446	0.382597	0.507466	<0.05
Number of foetuses	12.6	11.1	10.9	>0.05
Foetus birth weight (g)	1.97	1.64	1.69	>0.05
Placental weight (g)	1.46	1.38	1.48	>0.05
External malformations	0	1	0	>0.05

All data are presented as the mean ± SD.

a Analysed by one‐way analysis of variance.

### 3.2 RE ameliorates the epithelial characteristics of the placenta in PE model

**Figure 2:**
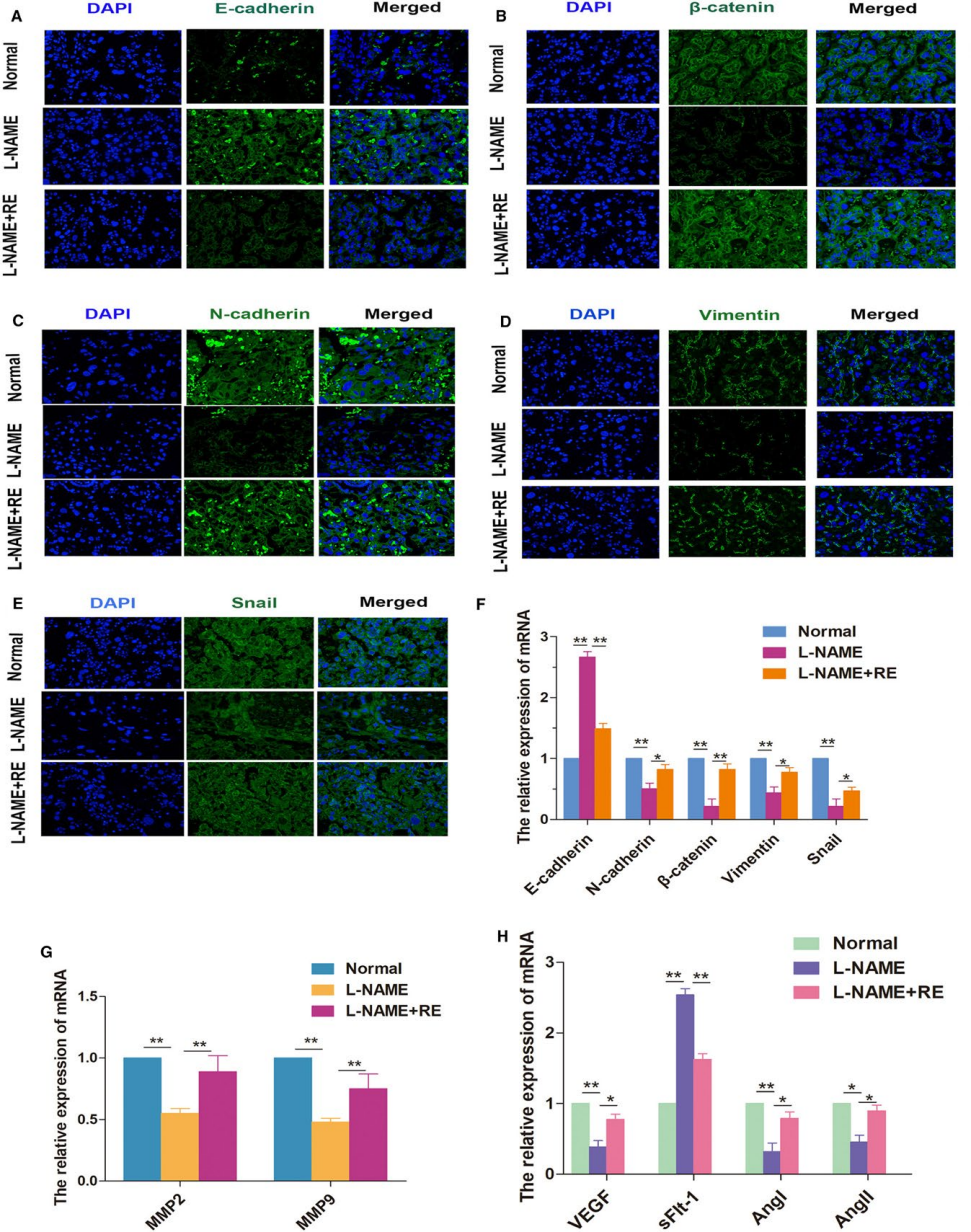
RE ameliorates the epithelial characteristics of the placenta in PE model. L‐NAME activated EMT in the mice placenta. A‐E, Immunofluorescence analysis of E‐cadherin, β‐cadherin, N‐cadherin, vimentin and snail expression, respectively, in the placenta of mice treated with L‐NAME or L‐NAME + RE. F, qRT‐PCR analysis of E‐cadherin, β‐cadherin, N‐cadherin, vimentin and snail expression in the placenta. G, qRT‐PCR analysis of MMP‐2 and MMP‐9. H, qRT‐PCR analysis of the angiogenesis‐related factors VEGF, sFlt‐1, AngI and AngII. All experiments were performed in triplicate with three technical replicates. *P < 0.05, **P < 0.01

So far, aberrant trophoblast invasion in placenta was recognized that was associated with EMT process and could cause the impaired spiral arteries remodelling, and further promote the progression of PE. Then, we wondered whether RE regulated EMT to ameliorate effects of RE on PE model. So, we performed the immunofluorescence assays and qPCR assays to detect the expression of the EMT‐related markers E‐cadherin, β‐cadherin, N‐cadherin, vimentin and snail, as well as the invasion indicators MMP‐2 and MMP‐9 (Figure 2A‐E). Our resulting data demonstrated that in the L‐NAME + RE group, E‐cadherin expression was lower, whereas β‐cadherin, N‐cadherin, vimentin, snail and MMP‐2/MMP‐9 levels were higher than those in the L‐NAME group (Figure 2F and G), further proving the role of RE in the activation of EMT process. Simultaneously, the levels of the angiogenesis‐related factors VEGF, sFlt‐1, AngI and AngII in PE rats were significantly improved by RE (Figure 2H). These results indicated the ability of RE in promoting the invasive capacity of trophoblasts, in partly through EMT activation, and thus RE increased the angiogenesis potential in the placenta of PE model.

### 3.3 Effect of RE on the migration and invasion in vitro

**Figure 3:**
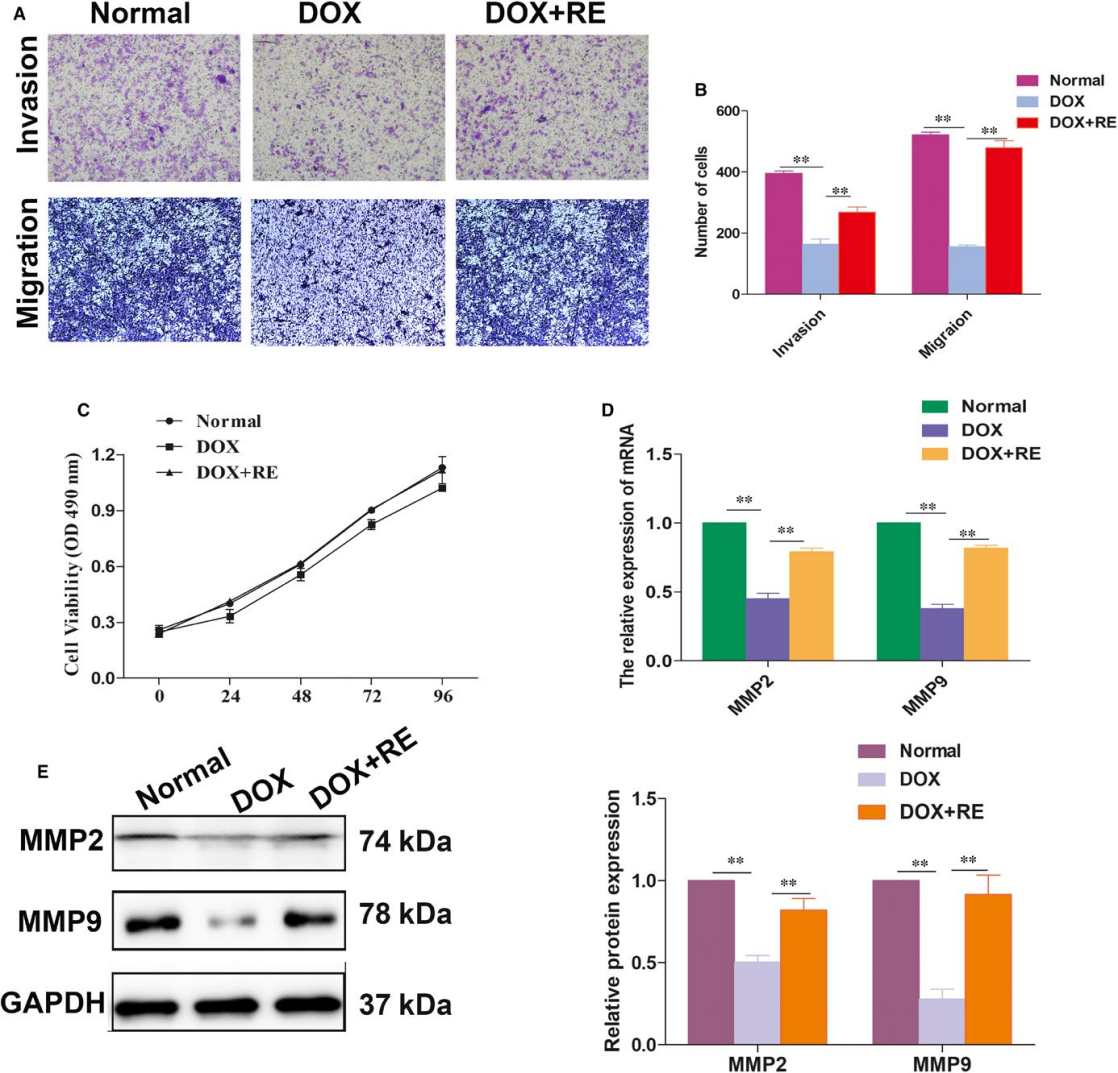
Effect of RE on the migration and invasion of trophoblast cells. A and B, The transwell assay was conducted to investigate the changes in migratory and invasive abilities of HTR‐8/SVneo cells. The migration and invasion capacities of the cells transfected with DOX were significantly lower than that of the normal and DOX + RE groups (data are presented as mean ± SD; **P < 0.01). C, There were no significant differences among the three groups, as observed using the MTT assay. D and E, qRT‐PCR and Western blotting analysis of MMP‐2 and MMP‐9 expression levels. **P < 0.01

In conjunction with the results of in vivo study, we choose trophoblast cells to further invalidate the signalling pathway in vitro. Here, to unravel whether RE regulates the EMT in trophoblast cells, we first selected RE and doxorubicin (DOX, a kind of EMT inhibitor^26^) to treat HTR‐8/SVneo respectively. After HTR‐8/SVneo cells were treated with RE, the capacity of cell migration and invasion, and proliferation were detected. As shown in Figure 3A and B, we conducted transwell assays and revealed that cells in the group treated with RE could significantly promote trophoblast migration and invasion compared with those in control. Moreover, we further confirmed that protein levels of MMP‐2 and MMP‐9 were significantly higher in the RE‐treated group than those in the control by qRT‐PCR and Western blotting assays (Figure 3D and E). In addition, MTT assays were conducted to examine the effect of RE on cellular growth activity. As shown in Figure 3C, there was no significant difference in cell growth activity between the RE‐treated and control groups (*P* > 0.05).

### 3.4 RE promoted invasion and tube formation by EMT in vitro

**Figure 4:**
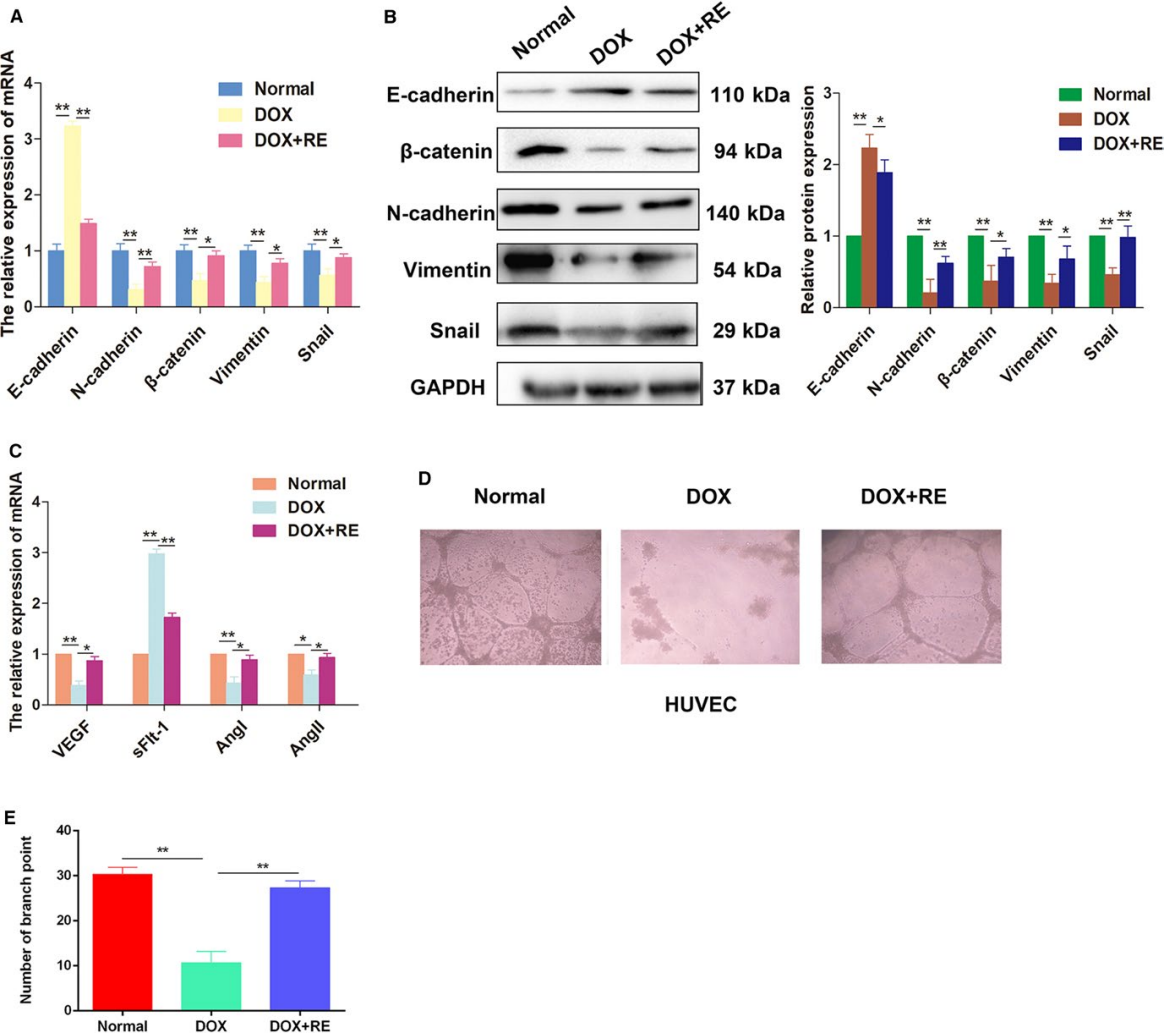
RE regulated EMT in HTR‐8/SVneo cells and affected the network formation ability of HUVEC. A and B, Related EMT biological markers expression in HTR‐8/SVneo cells, measured by qRT‐PCR and Western blotting. C, Expression of endothelial growth factors VEGF, sFlt1, AngI and AngII measured by qPCR after treatment with DOX or DOX + RE. D and E, Effects of DOX on cellular network formation ability in vitro. Cells in the DOX + RE group showed a higher node number than the DOX group. *P < 0.05, **P < 0.01

Our previous work has reported that the EMT was involved in trophoblast cell invasion and migration.^27^ qRT‐PCR assays were performed to detect the expression of EMT biomarkers. As shown in Figure 4A, the DOX+RE group showed elevated levels of the mesenchymal biomarkers N‐cadherin, β‐cadherin, vimentin and snail, as well as a decreased level of the epithelial biomarker E‐cadherin. Moreover, these observations were further confirmed by Western blotting (Figure 4B, *P* < 0.05). In addition, the number of branch point was significantly higher in trophoblasts treated with RE than that of the control, indicating a significant increase network formation ability (Figure 4D and E). The expression of the angiogenesis‐related factors VEGF, sFlt1, AngI and AngII was also higher in the DOX+RE group than those in the DOX group (Figure 4C, *P* < 0.05). Taken together, these findings suggested that RE could ameliorate cell invasion and tube formation by promoting the EMT process.

### 3.5 Effects of RE on the WNT/β‐catenin pathway

**Figure 5:**
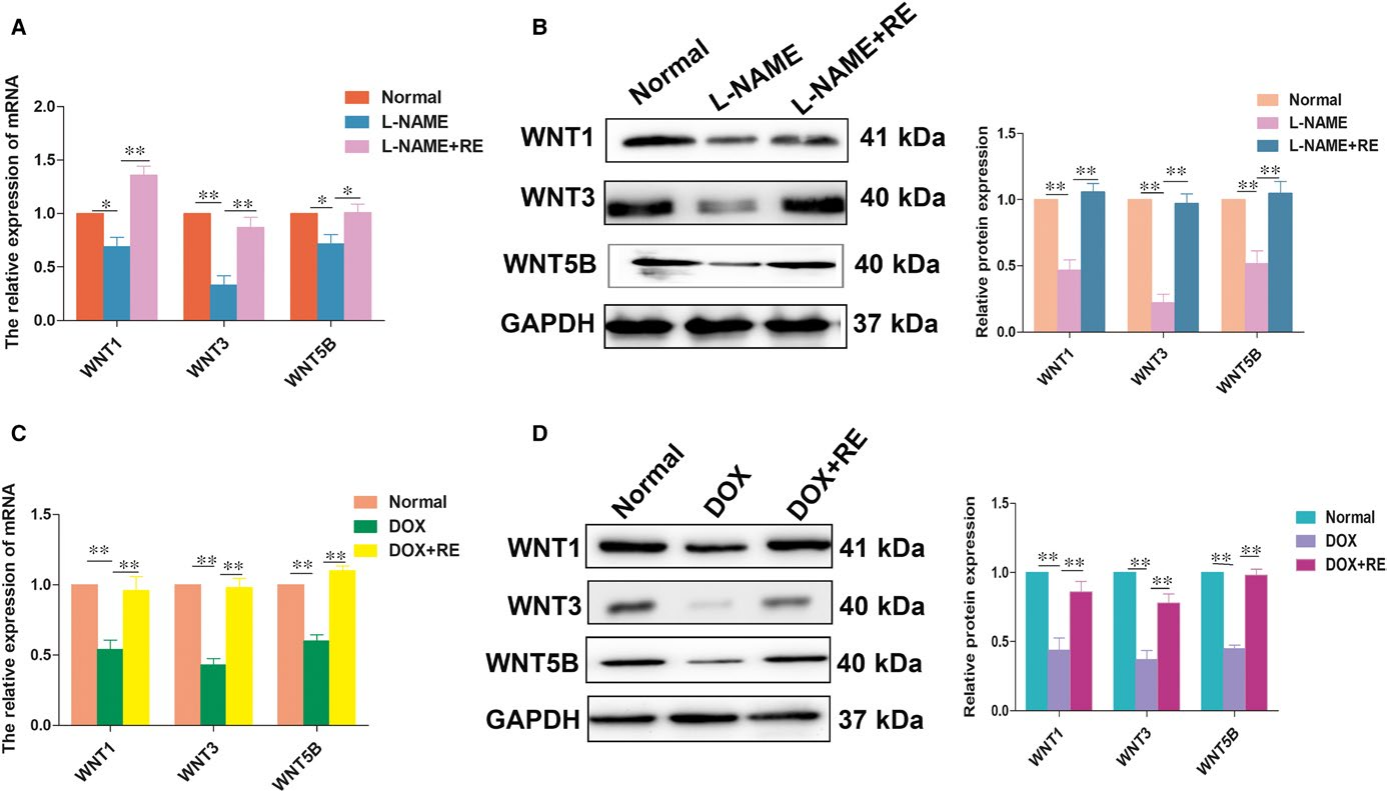
RE played a role in the Wnt/β‐catenin pathway. WNT1, WNT3 and WNT5B expression in mouse placenta (A and B) and HTR‐8/SVneo cells (C and D) measured by qRT‐PCR and Western blotting analysis. *P < 0.05, **P < 0.01

Previous studies have reported that β‐catenin is the key transcriptional activator in canonical Wnt signalling in the nucleus, and L‐NAME represses Wnt/β‐catenin‐mediated transcriptional activity by promoting the cytoplasmic localization of β‐catenin.^28^ In our study, we have validated that RE could ameliorative PE clinical phenotypes which stimulated by L‐NAME in mice model. So the effects of RE on Wnt‐related gene expression were detected in vitro and in vivo. We evaluated the mRNA levels of members of the WNT family that have been reported to be associated with EMT, including WNT1, WNT3 and WNT5B. The relative expression of WNT1, WNT3 and WNT5B mRNA decreased by 31%, 60% and 25%, respectively, following L‐NAME injection, and they increased by 2.2‐, 4.6‐ and 1.9‐fold, respectively, following RE treatment (Figure 5A) in PE mice model. Additionally, we observed the same changes in WNT1, WNT3 and WNT5B expression in the Western blotting results, as the same to qPCR results (Figure 5B). WNT1, WNT3 and WNT5B levels in the HTR‐8/SVneo cells treated with DOX+RE were different than in cells treated with DOX only, following a similar trend to that observed in the in vivo experiment (Figure 5C and D). Hence, these results suggest that RE might stimulate the invasive capability of human trophoblasts by mediating the Wnt/β‐catenin pathway.

## 4 DISCUSSION

Based on its great harm to pregnant women and perinatal infants, PE has attracted much attention in the clinical field. PE may be caused by placental ischaemia, vascular endothelial injury, poor immune adaptation, oxidative stress and genetic susceptibility,^17^ further suggested that placental ischaemia induces superficial placentation or the formation of a defective placenta in the development of the placental vascular bed. Extravillous cytotrophoblast (EVT) in the uterine wall is limited to the superficial parts of the uterine vascular invasion or incomplete invasion.^18^

Epithelial‐mesenchymal transition process is generally associated with the pathogenesis and progression of various humans disorders, included PE.^19^ A decrease in E‐cadherin expression is a fundamental event in EMT, and the decreased and increased expression of E‐cadherin and non‐epithelial cadherins (N‐cadherin), respectively, are pivotal indicators of EMT.^21^ Moreover, snail, the zinc‐finger transcription factor which represses the transcription of E‐cadherin, is thought to act as a key regulator of EMT.^21^ Previous studies have suggested that placental trophoblasts change from a coherently attached phenotype to a migratory phenotype capable of invading maternal decidua and spiral arteries in a process that resembles other developmental EMT. Therefore, we need to elucidate the regulatory mechanism of the programmed role of EMT in PE.

In our report we explored the role of RE in trophoblast both in vivo and in vitro. Hypertension and proteinuria were markedly improved after injection of RE compared with the controls in PE mice model. MTT and transwell assays were performed to further establish the various regulatory roles of RE in trophoblasts. And we revealed that RE improved cell migration and invasion but mildly and insignificantly improved cell viability. Moreover, RE could significantly increase the expression of MMP‐2 and MMP‐9 at mRNA and protein level, consistent with the results of the transwell assay. Also, RE could promote EMT process through the regulation of E‐cadherin, β‐catenin, N‐cadherin, vimentin expression by qPCR and Western blot assays. RE was involved in controlling the remodelling of spiral arteries by evaluating the angiogenesis‐promoting ability of RE in HUVEC and showed that RE ameliorated the effects of DOX and improved the network formation ability of HUVEC. RE considerably increased the number of capillary‐like networks and the expression of VEGF, sFlt1, AngI and AngII in vitro. Therefore, RE may promote angiogenesis and play an essential role in the remodelling of spiral arteries in PE mice model.

β‐catenin is the key transcriptional activator in the canonical Wnt signalling pathway in the nucleus, and RE increases Wnt/β‐catenin‐mediated transcriptional activity by promoting the nucleus localization of β‐catenin.^22^, ^29^ Then, we further analysed the related expression level of WNT1, WNT2 and WNT5B. These results, combined with the RE‐induced change in WNT1, WNT2 and WNT5B expression in HTR‐8/SVneo, indicated that RE may regulate EMT process in trophoblasts through the Wnt/β‐catenin signalling.

Thus, our study provides new insights into the biological mechanisms of RE in PE, in which RE might stimulate the invasive capability of human trophoblasts by promoting EMT process, partially by mediating Wnt/β‐catenin signalling pathway, to further impede the occurrence and development of PE.

## CONFLICTS OF INTEREST

The authors declare no conflict of interest.
